# Diverse Profiles of Toll-Like Receptors 2, 4, 7, and 9 mRNA in Peripheral Blood and Biopsy Specimens of Patients with Celiac Disease

**DOI:** 10.1155/2018/7587095

**Published:** 2018-07-02

**Authors:** Haniye Ghasiyari, Mohammad Rostami-Nejad, Davar Amani, Kamran Rostami, Mohamad Amin Pourhoseingholi, Hamid Asadzadeh-Aghdaei, Mohammad Reza Zali

**Affiliations:** ^1^Basic and Molecular Epidemiology of Gastrointestinal Disorders Research Center, Research Institute for Gastroenterology and Liver Diseases, Shahid Beheshti University of Medical Sciences, Tehran, Iran; ^2^Department of Immunology, School of Medicine, Shahid Beheshti University of Medical Sciences, Tehran, Iran; ^3^Gastroenterology and Liver Diseases Research Center, Research Institute for Gastroenterology and Liver Diseases, Shahid Beheshti University of Medical Sciences, Tehran, Iran; ^4^Department of Gastroenterology, Milton Keynes University Hospital, Milton Keynes, UK

## Abstract

**Background and Aims:**

Both adaptive and innate immunity are involved in the development of celiac disease (CD). Altered Toll-like receptors (TLR) expression and activation may be partially responsible for the inflammation and subsequently crypt hyperplasia, but the main driver for inflammation is gliadin-reactive T-cells. Therefore, the aim of this study was to investigate the TLRs 2, 4, 7, and 9 gene expressions in both peripheral blood and intestinal mucosa of patients with celiac disease compared to healthy control (HC).

**Material and Methods:**

Blood samples from 120 confirmed active CD patients and 120 age- and sex-matched healthy volunteers served as control group were collected during 2015-2016. Also, 20 biopsy specimens from the study group were randomly collected. Total RNA was isolated using a standard commercial kit. The mRNA expression of TLRs was quantified by relative qPCR with *β*2 microglobulin (*β*2m) as a reference gene.

**Results:**

TLR4 (*P* = 0.01) and TLR9 (*P* = 0.02) mRNA were significantly elevated in blood samples from CD patients compared to the healthy controls. Moreover, TLR2 (*P* = 0.03) and TLR4 (*P* = 0.0003) expression level was increased in CD biopsy specimens compared to controls, whereas expression of TLR9 mRNA was significantly decreased in CD patients. There was no significant difference in the expression of TLR7 in biopsy and blood specimens.

**Conclusions:**

The alteration of TLR4 and TLR9 expression in the blood and biopsy samples of patients with CD supports the critical role of the innate immune system in the pathogenesis of this disease. Upregulation of TLR4 and TLR9 suggests the contribution of gut microbiota or dysregulation of the immune response to commensal flora in small bowel mucosa in celiac patients.

## 1. Introduction

Celiac disease (CD) is defined as a chronic immune-mediated enteropathy characterized by aberrant immune response to ingested gluten and related proteins [1–3]. Most of the CD patients (≅98-99%) carry either DQ2 or DQ8 (or both) specific human leukocyte antigen (HLA) class II alleles [4, 5]. The clinical manifestations of the disease are highly variable in CD patients and include a wide range of GI and non-GI signs and symptoms [2, 6]. The diagnosis of celiac disease is based on changes in the histology of the small intestinal mucosa which is characterized by increased intraepithelial lymphocytes, crypt hyperplasia, villous flattening, and positive serological tests for antitransglutaminase antibodies (tTG) and/or endomysial autoantibodies (EMA) [2, 7].

Current knowledge emphasizes that the imbalance in the innate immune system contributes to the pathogenesis of CD [8, 9]. The imbalance seems to affect the small intestine of CD patients but not healthy individuals. The innate immunity is potentially activated by products derived from gut microbiota that are detected in the small intestine of some untreated and treated patients with CD [9, 10]. These products are recognized by Toll-like receptors (TLRs), which belongs to the group of pattern recognition receptors (PRRs) [11–13]. In addition, various components of TLRs initiate signaling cascades that are potent inducers of inflammatory cytokine productions such as IL-6, TNF*α*, and IFN in response to microbial and/or viral stimuli which may promote the development of autoimmune processes [12, 14].

Nevertheless, the main function of TLRs in the immunopathogenesis of CD has not yet been addressed, but available literature has shown that activating their signaling pathway induces the secretion of major histocompatibility complex, proinflammatory cytokines, chemokines, and recruitment of myeloid cells [8, 10, 15]. The inflammatory cascade caused by these factors leads to histopathological changes of the intestine (crypt hyperplasia and flattening of the intestinal villi) [16].

On the other hand, TLRs are able to increase intestinal barrier permeability and facilitate passage of gluten peptides and intestinal pathogens into the lamina propria. The myeloid differentiation factor 88 (MyD88), a key adapter molecule in TLRs signaling pathway is essential for this function [17].

Also, some previous studies have shown that TLR2 mRNA expression is increased in antigen-presenting cells (APCs) in CD, reflecting locally increased expression of TLR2 receptor already observed in CD, while the effects on TLR4 expression are more controversial [18, 19]. In other words, the alteration of TLR2 and TLR4 mRNA expression in the duodenal mucosa of patients with CD may indicate their potential association with CD [18].

In the present study, we hypothesized that there is a possible association between the TLR gene expression and progression of celiac disease. Few reported data are available about the expression and clinical relevance of TLRs in CD pathophysiology; therefore, the aim of this study was to investigate the TLRs 2, 4, 7, 9 gene expression in both peripheral blood and intestinal mucosa of patients with celiac disease compared to healthy control (HC).

## 2. Materials and Methods

### 2.1. Study Population

This is a part of a cross-sectional study that abstracted data from patients' documents during the period May 2015–May 2016. One hundred and twenty CD patients (mean age 34.50 ± 14.9 years) and 120 healthy controls (mean age 40.40 ± 15.6 years) referred to the Research Institute for Gastroenterology and Liver Diseases, Shahid Beheshti University of Medical Sciences (Tehran, Iran) were included. For each healthy subject and CD patient, a verified questionnaire including demographic data and clinical and histological findings was completed and information about GI and non-GI symptoms of patients was recorded. Also, 8–10 ml peripheral blood samples were collected and stored at −70°C until DNA/RNA extraction.

Among the CD patients, 20 duodenal biopsy samples were randomly collected. Each patient had a confirmed diagnosis based on celiac serology markers (anti-tissue transglutaminase antibodies and/or anti-endomysium antibodies), standard endoscopic and histology approval according to the original Marsh classification [20]. Also, the frequency of GI, non-GI symptoms, and histological abnormalities was reported. Control group was selected from those who undergone endoscopy and biopsy with indications like dyspepsia and anaemia showing normal histology. Patients and controls with history of *Helicobacter pylori* infection, autoimmune disorders such as IBD, type one diabetes, and GI infection (viral and bacterial) were excluded from the study.

### 2.2. Ethical Considerations

Written informed consent was obtained from all of the study subjects. The study adheres to national regulations and was approved by the ethics committee of Research Institute for Gastroenterology and Liver Diseases, Shahid Beheshti University of Medical Sciences (IR.SBMU.RIGLD.1395.88).

### 2.3. RNA Extraction and Quantitative Real-Time PCR

The RNA was extracted from white blood cells using commercial Kit (RNA Extraction kit, Yekta Tajhiz Azma, Tehran, Iran) according to the manufacturer's instructions. In addition, biopsy samples first were washed with RNAase-free water and then immediately submerged in RNA later (Pars tous, Tehran, Iran). Samples were stored at −70°C until RNA isolation. The rest of the RNA extraction steps were performed in the same way as blood samples.

Isolated RNA was eluted to a new RNAase-free tube with 60 *μ*l of RNAase-free water and stored at −70°C. RNA concentration was measured by NanoDrop ND-1000 spectrophotometer (NanoDrop Technologies, Wilmington, USA), and the quality of RNA was analyzed with Bio-Rad Experion System (Bio-Rad Laboratories, Hercules, CA, USA). The cDNA was synthesized using a Revert Aid RT Reverse Transcription kit (cat. number K1691; Thermo Fisher Scientific, Inc.) according to the manufacturer's instructions. In reverse transcription reactions, only high-quality RNA was used.

Primers were designed using GenScript online programs (https://www.genscript.com/) for investigated TLRs as follows: Forward, 5- GCATGTGGTGTCCTCTGTTC -3 and reverse, 5- GAGCTTTCCTTGGCCTCCTT -3 for TLR2, Forward 5- TGGACCTGCGATTTAATCCC 3- and reverse, 5- GTCTGGATTTCAGAGCAGGA -3 for TLR4, Forward 5- CACCTGTAGTGCTGTGTCGTT -3 and reverse, 5- TCACATCTGAGGGCACCTAAG -3 for TLR7, Forward 5- AGGCACCTGTCACTCTTGTACA -3 and reverse, 5- GTAGGACAACAGCAGACGATCC -3 for TLR9, Forward 5- TGCTGTCTCTGAGTTTGATGTATCT-3 and reverse, 5- TCTCTGCTCCCCACCTCTATAG -3 for *β*2m. The expression levels of all target genes were normalized against the expression of *β*2m, which served as the endogenous control due to its invariable expression in all of the samples used in the study.

The expression rate of the TLRs mRNA was analyzed using an ABI 7500 Real-Time PCR System (Applied Biosystems, USA) and SYBR Master Mix (Takara Bio, Inc., Otsu, Japan) according to the manufacturer's instructions. Reactions were run in a total volume of 20 *μ*l with 1 *μ*l of cDNA in each and 10 *μ*l SYBR Green Master Mix with the following cycling conditions: 95°C for 30 sec, 40 cycles of 95°C for 5 sec, and 60°C for 34 sec. The melting curve which differentiates between the different products demonstrates contamination and the absorption peak. Melting curve analysis also detects nonspecific and primer dimer products. The relative expressions of genes were calculated according to the ΔΔCt method. SDS RQ Manager Program (Applied Biosystems, version 2.3) was used to analyze the results.

### 2.4. HLA Typing

For HLA typing, genomic DNA was extracted using salting out method and DQ2/DQ8 haplotypes were genotyped by real-time PCR using SYBR Green as described previously [21].

### 2.5. Statistical Analysis

SPSS statistical software version 20 (IBM Corp., Armonk, NY, USA) was used to perform statistical analysis of the demographic data and clinical and histological findings. Comparison of variables was performed using chi-squared, Spearman's correlation, and one-way ANOVA tests.

GraphPad Prism 6.0 software (https://www.graphpad.com/scientific-software/prism/) was used for the statistical analysis of TLR mRNA expression levels. Data involving two groups were compared using the Student's *t*-test. A *P* < 0.05 was considered as statistically significant. Data were expressed as mean and standard deviation (mean ± SD).

## 3. Results

### 3.1. Demographic Data

A total of 120 patients (mean age of 34.5 ± 14.9) including 38 males (31.6%) and 82 females (68.3%) with CD were investigated. The control group comprised 120 non-CD subjects (mean age of 40.4 ± 15.6) including 46 males (38.3%) and 74 females (61.6%). There was no significant difference between genders in the two groups (*P* = 0.74).

The most common GI symptoms in CD patients were diarrhea (56.6%), bloating (51.6%), and weight loss (42%). Also, among extraintestinal manifestations, aphthous (46.6%), anemia (35.8%), and growth failure (28%) were the most common presentations (Table 1). Pathologic reports were Marsh I in 22.4%, Marsh II in 12.2%, and Marsh III in 65.3%. HLA typing was performed, and in 120 CD patients, HLA DQ2 was positive in 71 cases (59.1%), DQ8 in 28 (23.3%), −DQ2/8 in 15 (12.5%), and 6 (5%) were negative. In 120 control, DQ2 was positive in 32 cases (27%), DQ8 in 15 (12.5%), −DQ2/8 in 6 (5%), and 67 (55.9%) were negative.

In the present study, we investigated the expression levels of TLR2, TLR4, TLR7, and TLR9 in peripheral blood and duodenal biopsy of patients with CD compare to controls.

### 3.2. Blood Samples

**Figure 1:**
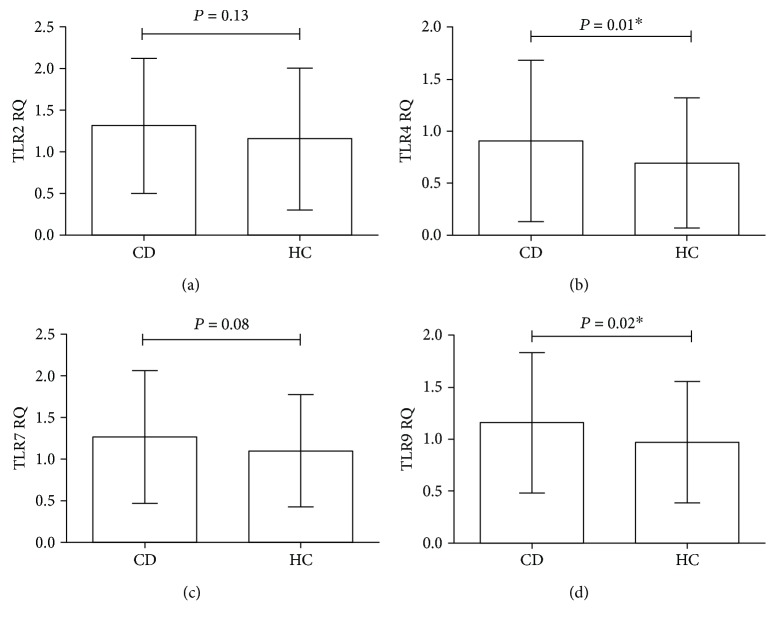
The relative expression of Toll-like receptors (TLRs) mRNA in peripheral blood specimens. Analysis of significance was performed by Student's *t*-test. (a) TLR2, (b) TLR4, (c) TLR7, and (d) TLR9. ^∗^*P* value is significant.

The results showed that there is no statistically significant difference of TLR2 mRNA expression between the CD cases and the healthy controls (CD: mean ± SD: 1.31 ± 0.07; healthy controls: mean ± SD: 1.15 ± 0.07; *P* = 0.13) (Figure 1(a)). However, the TLR4 mRNA expression was significantly increased in blood of CD patients compared to controls (CD: mean ± SD: 0.91 ± 0.07; healthy controls: mean ± SD: 0.69 ± 0.05; *P* = 0.01) (Figure 1(b)). Similar to TLR2 mRNA expression, the TLR7 gene expression was not statistically different between the groups (CD: mean ± SD: 1.26 ± 0.07; healthy controls: mean ± SD: 1.09 ± 0.06; *P* = 0.08) (Figure 1(c)), whereas mRNA expression of TLR9 was significantly increased in CD patients compared to controls (CD: mean ± SD: 1.15 ± 0.06; healthy controls: mean ± SD: 0.97 ± 0.05; *P* = 0.02) (Figure 1(d)).

### 3.3. Biopsy Specimens

**Figure 2:**
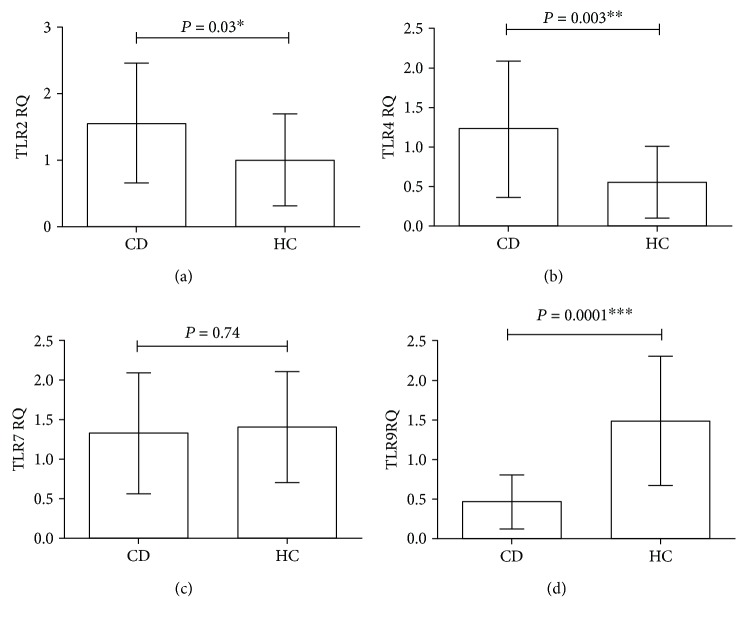
The relative expression of Toll-like receptors (TLRs) mRNA in small intestinal biopsies. Analysis of significance was performed by Student's *t*-test. (a) TLR2, (b) TLR4, (c) TLR7, and (d): TLR9. ^∗^, ^∗∗^, ^∗∗∗^*P* value is significant.

The mRNA expressions of investigated TLRs in the duodenal mucosa of representative 20 samples from CD patients and 20 healthy control are shown in Figure 2. In contrast to blood samples, we found even significantly higher TLR2 mRNA level in the duodenal mucosa of the patient group compare to controls (CD: mean ± SD: 1.55 ± 0.20; healthy controls: mean ± SD: 1.00 ± 0.15; *P* = 0.03) (Figure 2(a)). TLR4 gene expression was higher than TLR2 mRNA expression. Our results showed a statistically significant increased expression of TLR4 in CD subjects compared to controls (CD: mean ± SD: 1.22 ± 0.19; healthy controls: mean ± SD: 0.55 ± 0.10; *P* = 0.003) (Figure 2(b)). However, similar to the gene expression of TLR7 in the blood, no statistically significant difference was detected between the cases and controls (CD: mean ± SD: 1.32 ± 0.17; healthy controls: mean ± SD: 1.40 ± 0.15; *P* = 0.74) (Figure 2(c)). Interestingly, mRNA expression of TLR9 was significantly decreased in CD patients compared with controls (CD: mean ± SD: 0.46 ± 0.07; healthy controls: mean ± SD: 1.48 ± 0.18; *P* = 0.0001) (Figure 2(d)).

### 3.4. Clinicopathological Factors

We also evaluated the possible association between TLR gene expression and clinicopathological factors (including sex, HLA-DQ2/DQ8 expression, and degrees of histological abnormalities), in individuals with CD. Regarding the TLR gene expression and sex distribution in CD patients, the results showed that except for TLR4 (males: mean ± SD: 1.31 ± 0.79, females: mean ± SD: 1.09 ± 0.69; *P* = 0.02), there were no statistically significant associations between this factor and gene expression of TLR2 (males: mean ± SD: 1.26 ± 0.78, females: mean ± SD: 1.21 ± 0.86; *P* = 0.58), TLR7 (males: mean ± SD: 1.74 ± 0.70, females: mean ± SD: 1.83 ± 0.71; *P* = 0.98) and TLR9 (males: mean ± SD: 1.00 ± 0.61, females: mean ± SD: 1.09 ± 0.64; *P* = 0.62) in blood specimens. Similarly, no statistically significant associations were detected between sex distribution and TLR gene expression in control groups (TLR2 (males: mean ± SD: 1.24 ± 0.23, females: mean ± SD: 1.23 ± 0.31; *P* = 0.9), TLR4 (males: mean ± SD:1.21 ± 0.35, females: mean ± SD:1.08 ± 0.7; *P* = 0.32), TLR7 (males: mean ± SD: 1.6 ± 0.63, females: mean ± SD: 1.09 ± 0.27; *P* = 0.9), and TLR9 (males: mean ± SD: 1.04 ± 0.96, females: mean ± SD:1.54 ± 0.7; *P* = 0.07)).

Again, no statistically significant difference was detected between TLR gene expression and sex distribution in biopsy samples of cases and controls. However. Spearman's correlation test showed that there is a significant correlation only for TLR4 and severity of mucosal damages in blood sample (Spearman rho = 0.429; *P* = 0.03); in contrast, our analyses demonstrated the lack of no correlation between mucosal abnormalities and TLR 2, 7, and 9 in blood and biopsy specimens.

Regarding TLR gene expression and HLA-DQ2/DQ8 expression in both blood and biopsy specimens, there was no significant correlation (data not shown).

## 4. Discussion

In the present study, TLR mRNA level in peripheral blood and duodenal mucosa were investigated and compared with healthy controls. According to the results of this study, we found statistically significant upregulation of TLR4 (*P* = 0.01) and TLR9 (*P* = 0.02) in blood samples of CD compare to HC but not for TLR2 (*P* = 0.13) and TLR7 (*P* = 0.08). Moreover, in duodenal mucosa of CD patients, mRNA expressions of TLR2 (*P* = 0.03) and TLR4 (*P* = 0.003) were significantly increased, whereas TLR9 (*P* = 0.0001) expression was downregulated in patients compared to controls. There were also no significant differences in the expression of TLR7 (*P* = 0.74) in biopsy specimens.

Similar to the recent study by Brynychova et al. [22], we found that there was a significant difference of TLR4 but not for TLR2 mRNA in CD patients in peripheral blood compared to controls. However, in contrast to our study, they found that peripheral blood monocytes (PBMs) from patients with active CD expressed significantly higher TLR7 mRNA levels than CD-GFD subjects and healthy controls.

TLR7 is a nucleotide-sensing TLRs which binds to single-stranded RNA (ssRNA) from viruses [23]. Zanoni et al. [24] reported that sera obtain from celiac patients contain antibodies against a factor which was highly similar to a viral component. They suggest that some viral infections such as *rotavirus* infection, which causes gastroenteritis, through the mechanism of molecular mimicry helps to initiate celiac disease in susceptible individuals [24]. Furthermore, a study carried out by Kagnoff et al. [25] suggested that CD was initiated by *adenovirus*-12 infection. Therefore, it is possible that TLR7 is the mediator of this activation [26].

Increased gene expression level of TLR9 was demonstrated for the first time in peripheral blood samples in celiac patients compared to healthy controls. A possible elevation of this marker could be due to the upregulation of innate immune cell activation by bacterial components according to the loss of intestinal epithelial barrier [10, 27].

In this study, in addition to evaluating the expression of TLR genes in peripheral blood, we also examined their changes in small intestinal biopsies. Controversial reports have been published regarding the expression of TLRs in the small intestinal biopsies in patients with celiac disease in previous studies (Table 2).

For example, Szebeni et al. [18] found an increased expression of TLR2 and TLR4 and Eiro et al. [19] observed this significant raised of TLR4 in both untreated and treated CD patients comparing them to controls for both mRNA and protein levels. These reports are similar to our findings and bring more strength to the body of evidence supporting the role of these mediators in CD pathogenesis that these results confirmed our data. The evidence suggest that TLR4 may represent a new diagnostic marker and perhaps a good therapeutic target for CD [18], having said that there are controversial reports in the current literature. For instance, Kalliomaki et al. [10] reported that TLR2 was decreased in celiac patients compared to controls. The differences may be due to differences in patient selection and possibly the different methods of studies. In contrast to Kalliomaki et al. [10], in our study, a significant decrease in TLR9 mRNA expression in the biopsy samples was observed in CD patients compared to controls. They suggest that increased expression of the TLR9 mRNA in untreated CD may be either another function of the TLR9 gene or a defect in the signaling of the gene in these patients [10].

Reassuringly, Eiro et al. [19] have shown that no significant difference for TLR7 gene expression between CD and HC in duodenal mucosa.

In addition, Eiro et al. [19] did not find a significant association between immunohistochemical values, clinicopathological factors (including sex, HLA-DQ2/DQ8 expression, tissue transglutaminase (tTG) expression, and the histology), and TLR4 expression in the study population [19]. However, results of the present study indicate that there was a significant correlation between TLR4 expression and histological changes in blood specimens. In other words, increasing expression of TLR4 in the tissue samples may result in progressively a more severe higher mucosal damage. A significant difference for TLR4 according to the gender might be due to significantly higher levels of immune activation and expression of genes associated with inflammatory response in the female's gut microenvironment compared to men and different gut microbial communities between two sexes [28, 29].

CD may develop when mucosal or commensal homeostasis is impaired like an environmental trigger in patients with genetic predisposition [30]. Although the role of TLRs in the pathogenesis of celiac disease is not clear, it seems that pathogenic wheat components by activating the TLR pathway lead to some abnormal immune responses [8]. Dysfunction or aberrant signaling of TLR induces mucosal tissue damage or barrier wreck through stimulating the secretion of cytokines and chemokines and by the loss of commensal mediated colonic epithelial progenitor responses [12, 31, 32].

TLRs are responsible for maintaining a hyporesponsive state to the luminal content in the gastrointestinal tract. Intestinal luminal content is composed of the microbiota and the food ingredients [33, 34]. Also, data from the previous studies demonstrated that certain gliadin peptides such as p31-p43 dos not bind to HLA-DQ2 or HLA-DQ8 [17, 34] but are able to induce an immune response through TLRs [17, 34, 35]. Therefore, we suggest that the direct contribution of gliadin and/or other wheat components in activating TLR signaling. According to results of our study, it seems that the alteration of TLR4 and TLR9 expression in the blood and biopsy samples of active CD patients supports the critical role of the innate immune system in the pathogenesis of this disease. This may create a potential use of TLR in diagnostic and/or as prognostic markers and perhaps good therapeutic targets in CD. This will requires large-scale future studies to a thorough validation of similar studies for possible biomarkers in diagnostic and prognostic assessment.

One of the main limitations of the present study is that we could not evaluate the expression of these TLRs at the cell population and proteins levels, which would provide more relevant insight into their role in pathogenesis. We suggest that the determining protein level of TLRs by specific techniques such as Western blot would add great value in assessing these PRR pathways.

## 5. Conclusion

Based on the results of our study, we suggest that intestinal mucosa is the predominant site of TLRs expression, particularly for TLR2 and TLR4. Also, the different expression of TLR2, TLR4, and TLR9 in both blood and biopsy specimens may reflect the consequences of the interaction of intestinal epithelial cells with intestinal microflora involved in pathogenetic mechanisms of CD.

Since the innate immune system is the initiator of the immune responses against gluten and the potential role of TLR signaling in the pathogenesis of CD, investigation of protein-protein interaction network by coimmunoprecipitation assays, as well as small interfering RNA (siRNA) or TLR inhibitors, is suggested. In addition, the study of functional analysis of the effect of gliadin on TLR pathway and intestinal epithelium cells is recommended.

## Figures and Tables

**Table 1:** Frequency of gastrointestinal (GI) and non-gastrointestinal (non-GI) symptoms in the patient group at the admission.

Variable	*N* (%)
Diarrhea	68 (56.6)
Bloating	62 (51.6)
Weight loss	51 (42)
Nausea/vomiting	37 (30.8)
Constipation	16 (13)
Aphthous	56 (46.6)
Anemia	43 (35.8)
Stunting	34 (28)
Decreased bone age	31 (25.8)
Abortion	16 (13.3)
Infertility	11 (9.1)
Thyroid disease	3 (2.5)
Asthma	2 (1.6)

**Table 2:** Comparison of TLR gene expression between the present and previous studies in blood and biopsy specimens of patients with active CD.

Gene	Sample	Present study	Previous studies (ref)
TLR2	Blood	No significant difference of TLR2 mRNA in CD patients compared with HC.	No significant difference of TLR2 mRNA in CD patients compared with HC [22].
	Biopsy	Higher expression of TLR2 mRNA in CD patients compared with HC.	Higher expression of TLR2 mRNA in CD patients compared with HC [18].
			Decrease expression of TLR2 mRNA in CD patients compared with HC [10].

TLR4	Blood	Higher expression of TLR4 mRNA in CD patients compared with HC.	Higher expression of TLR4 mRNA in CD patients compared with HC [22].
	Biopsy	Higher expression of TLR4 mRNA in CD patients compared with HC.	Higher expression of TLR4 mRNA in CD patients compared with HC [18, 19].

TLR7	Blood	No significant difference of TLR7 mRNA in CD patients compared with HC.	Higher expression of TLR7 mRNA in CD patients compared with HC [22].
	Biopsy	No significant difference of TLR7 mRNA in CD patients compared with HC.	No significant difference of TLR7 mRNA in CD patients compared with HC [19].

TLR9	Blood	Higher expression of TLR9 mRNA in CD patients compared with HC.	Not investigated.
	Biopsy	Decrease expression of TLR9 mRNA in CD patients compared with HC.	Higher expression of TLR9 mRNA in CD patients compared with HC [10].

## Data Availability

The data used to support the findings of this study are available from the corresponding author upon request.
